# Comparative analysis of inflammatory markers as predictive markers for postoperative delirium in cardiac surgery patients: an observational study

**DOI:** 10.3389/fmed.2025.1515940

**Published:** 2025-04-01

**Authors:** Liang Shan, Keyang Zheng, Wenlong Dai, Yintang Wang, Peng Hao

**Affiliations:** ^1^Department of Cardiology, Beijing Anzhen Hospital, Capital Medical University, Beijing, China; ^2^Department of General Practice, Beijing Nuclear Industry Hospital, Beijing, China; ^3^Department of Cardiology, Beijing Tsinghua Changgung Hospital, School of Clinical Medicine, Tsinghua University, Beijing, China

**Keywords:** postoperative delirium, cardiac surgery, neutrophil-to-lymphocyte ratio, monocyte-to-lymphocyte ratio, platelet-to-lymphocyte ratio

## Abstract

**Background:**

Postoperative delirium (POD) is a common complication following cardiac surgery that significantly affects patient outcomes. Among inflammatory markers, the monocyte-to-lymphocyte ratio (MLR) has shown potential in predicting POD. However, studies on the relationship between neutrophil-to-lymphocyte ratio (NLR) or platelet-to-lymphocyte ratio (PLR) and POD are still lacking. Moreover, a direct comparison of the predictive capabilities of these three inflammatory markers (NLR, MLR, and PLR) for POD remains unexplored.

**Methods:**

This observational study utilized the MIMIC database. We included 2,095 patients who underwent cardiac surgery. Multivariable logistic regression analysis, restricted cubic spline (RCS) analysis, and receiver operating characteristic (ROC) curve analysis were employed to assess the relationship between NLR, MLR, PLR, and POD.

**Results:**

POD occurred in 415 patients (19.8%). Multivariable logistic regression identified NLR (OR 1.05, 95% CI 1.03–1.08), MLR (OR 1.39, 95% CI 1.01–1.92), and PLR (OR 1.00, 95% CI 1.00–1.00) as independent risk factors for POD, all with *P-*values < 0.05. ROC curve analysis revealed NLR had the strongest predictive ability (AUC = 0.610, 95% CI: 0.589–0.631), outperforming MLR (AUC = 0.575, 95% CI: 0.553–0.596) and PLR (AUC = 0.553, 95% CI: 0.531–0.574). RCS analysis indicated linear or near-linear relationships between these markers and POD risk.

**Conclusion:**

NLR, MLR, and PLR independently predicted postoperative delirium following cardiac surgery, with NLR demonstrating the strongest predictive capacity. These findings provided new tools for preoperative risk assessment and may improve postoperative management strategies for cardiac surgery patients.

## 1 Background

Postoperative delirium (POD) is an acute alteration in attention, awareness, and cognitive function, typically triggered by medical conditions and not explicable by pre-existing neurocognitive disorders (1). POD not only prolongs hospital stay and increases healthcare costs but may also affect patients' long-term cognitive function (2). This complication is particularly common among cardiac surgery patients, with meta-analyses indicating an incidence rate ranging from 2.9% to 54.9%, significantly higher than other major surgeries (3, 4). For cardiac surgery patients, a systematic review and meta-analysis revealed that POD is associated with multiple adverse outcomes, including increased mortality, prolonged ICU and hospital stays, and extended duration of mechanical ventilation (5). Given its high incidence and severe consequences, in-depth research into risk factors and preventive strategies for POD following cardiac surgery holds significant clinical importance.

The high incidence and severe consequences of POD following cardiac surgery have prompted researchers to delve deeper into its potential mechanisms, with inflammatory responses increasingly becoming a focus of attention. In recent years, neutrophil-to-lymphocyte ratio (NLR), monocyte-to-lymphocyte ratio (MLR), and platelet-to-lymphocyte ratio (PLR) have garnered widespread attention as emerging inflammatory markers (6–8). These indicators can be easily calculated from routine blood tests without the need for additional complex or expensive examinations (9), and are considered reliable indicators of immune system homeostasis and disease progression (10). As cost-effective and readily available biomarkers, NLR, MLR, and PLR have been widely applied in clinical practice (11). Multiple studies have demonstrated that these indicators showed good prognostic predictive ability in neurological diseases. For instance, NLR was closely associated with the initial severity and short-term functional prognosis of acute ischemic stroke patients, with higher NLR indicating poorer outcomes (12). PLR, beyond its applications in stroke, has also been shown to be related to the risk of progression in mild cognitive impairment with higher PLR potentially indicating faster cognitive decline (13). MLR has also shown promise in neurodegenerative diseases, with a prospective study finding that elevated MLR levels were associated with accelerated cognitive decline in Alzheimer's disease patients (14). However, in the field of POD following cardiac surgery, existing studies primarily focused on the evaluation of single indicators. Only one study identified MLR as a risk factor for POD after cardiac surgery (15). Nevertheless, there was a lack of attention to the relationship between NLR or PLR and POD in this context. Moreover, a direct comparison of the predictive efficacy of these inflammatory markers (NLR, MLR, and PLR) for POD following cardiac surgery was still missing.

This study aimed to comprehensively evaluate the relationship between three inflammatory markers (NLR, MLR, and PLR) and the occurrence of POD following cardiac surgery, while also conducting a direct comparison among them. Through this in-depth investigation, we expect to thoroughly explore these inflammatory indicators and identify the most predictive factors. This will contribute to the development of effective preventive strategies, ultimately improving patients' postoperative outcomes.

## 2 Methods

### 2.1 Study population

This study employed a retrospective design and adhered strictly to the ethical principles of the Declaration of Helsinki. We obtained research data from the Medical Information Mart for Intensive Care IV (MIMIC-IV) database. MIMIC-IV is a comprehensive and publicly available clinical database containing detailed clinical information of patients hospitalized at Beth Israel Deaconess Medical Center (BIDMC) between 2008 and 2019. The database encompasses various aspects of patient data, including length of stay, laboratory test results, medication regimens, vital signs, and other relevant clinical information (16). To protect patient privacy, all patient identifiers were replaced with random codes, thus eliminating the need for individual consent or additional ethical approval.

**Figure 1:**
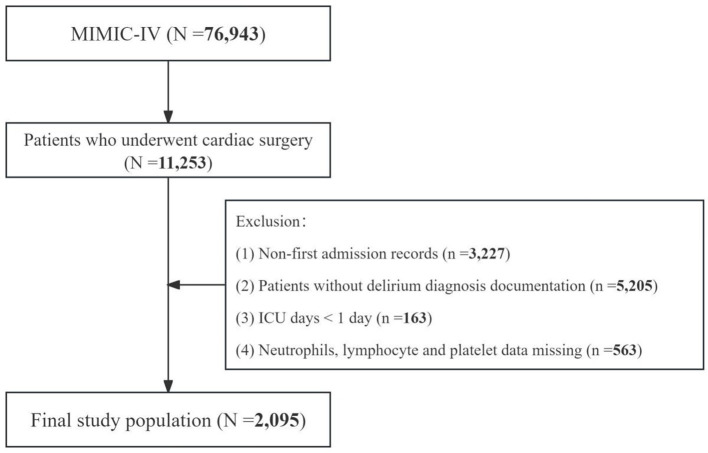
Flow diagram of the patient selection process.

This study initially encompassed 76,943 patients from the MIMIC-IV database. A rigorous selection process, adhering to predefined inclusion and exclusion criteria, was employed to determine the final study cohort. This study included individuals who underwent cardiac procedures during their hospital stay, as identified by the International Classification of Diseases, Ninth or Tenth Revision (ICD-9/10) codes (*n* = 11,253). All cardiac surgery-related ICD codes are provided in Supplementary Table S1. Subsequently, exclusion criteria were applied as follows: (1) non-first admission records (*n* = 3,227); (2) absence of delirium diagnosis documentation (*n* = 5,205); (3) hospital length of stay < 24 h (*n* = 163); and (4) incomplete hematological data, including neutrophil count, lymphocyte count, platelet count, and monocyte count (*n* = 563). Following this meticulous screening process, a total of 2,095 patients met the eligibility criteria and were included in the final analysis. The patient selection algorithm is graphically represented in Figure 1.

### 2.2 Data collection

Relevant clinical data were extracted from the MIMIC-IV database using Structured Query Language (SQL). The extracted data encompassed demographic characteristics, vital signs, key laboratory parameters, and major comorbidities. Demographic variables included age, sex, ethnicity, and marital status. Vital signs comprised systolic blood pressure (SBP) and diastolic blood pressure (DBP), heart rate, respiratory rate, and body temperature. Laboratory parameters primarily consisted of hematological indices, hepatic and renal function markers, and electrolyte levels. Furthermore, we documented major comorbidities potentially associated with cardiac surgery and POD, including but not limited to hypertension, diabetes mellitus, congestive heart failure, and atrial fibrillation. Types of cardiac surgery were also recorded to account for surgical variation. The primary outcome measure was the occurrence of POD. For all variables, we utilized the first recorded values following patient admission. For all variables, we utilized the first recorded values following patient admission. This standardized approach was chosen to ensure consistency in data collection timing across all study participants and to capture baseline patient characteristics before surgical intervention. For laboratory parameters specifically, all values were obtained from the same initial blood sample to minimize temporal variations in measurements.

### 2.3 Grouping and outcome

For baseline characteristics comparison, patients were stratified into two cohorts based on the presence or absence of POD during hospitalization. For multivariable regression analysis, patients were categorized into quartiles (Q1–Q4) according to their NLR, MLR, and PLR values, respectively. NLR was calculated as the ratio of neutrophil count to lymphocyte count, MLR as the ratio of monocyte count to lymphocyte count, and PLR as the ratio of platelet count to lymphocyte count (7, 17, 18).

The outcome of this investigation was the occurrence of POD during the hospital stay. For patients included in the MIMIC-IV database, delirium was screened through the confusion assessment method for the intensive care unit (CAM-ICU) and diagnosed with the Diagnostic and Statistical Manuals of Mental Disorders (DSM-5). The following ICD codes were used to identify POD cases: ICD-9 codes (29281, 2930, 2931, 2939, 34831, 34982, 78009, 78097) and ICD-10 codes (F05, G92, G9341, R410, R4182). The diagnostic spectrum encompassed various clinical manifestations including altered mental status, delirium due to secondary conditions, disorientation, drug-induced delirium, metabolic and toxic encephalopathy, consciousness alterations, subacute delirium, and transient mental disorders.

### 2.4 Statistical analysis

Statistical analysis was performed using R software (version 4.3.2, http://www.R-project.org). Continuous variables were assessed for normality using the Shapiro-Wilk test. Normally distributed variables were presented as mean ± standard deviation and compared using one-way ANOVA, while non-normally distributed variables were expressed as median (interquartile range) and compared using the Kruskal-Wallis H test. Categorical variables were presented as frequencies (percentages) and compared using chi-square or Fisher's exact tests. To investigate the associations between NLR, MLR, PLR and POD, multivariable logistic regression models were constructed. Covariates were selected based on previously established associations with POD, clinically significant differences in baseline characteristics between groups (*P* < 0.01), and clinical relevance. Model 1 was unadjusted; Model 2 adjusted for demographic factors; and Model 3 further adjusted for medical history and biochemical indicators. Inflammatory markers were analyzed both as continuous and categorical variables (Q1–Q4), reporting odds ratios (OR), 95% confidence intervals (CI), and *P-*values. Trend tests were conducted to assess linear trends between these indicators and POD risk. Restricted cubic spline (RCS) analysis was employed to explore potential non-linear relationships between NLR, MLR, PLR and the risk of POD. The predictive performance of these indicators for POD was evaluated using receiver operating characteristic (ROC) curve analysis, with areas under the curve (AUC) compared using the DeLong test. Subgroup analyses stratified by WBC count (≤ 10 × 10^9^/L and >10 × 10^9^/L) were performed to evaluate the potential impact of severe infections. The associations between inflammatory markers and POD were tested using logistic regression in each subgroup, with interaction terms added to test WBC count interactions. Statistical significance was defined as a two-tailed *p-*value < 0.05.

## 3 Results

### 3.1 Baseline characteristics of the participants

After screening based on inclusion and exclusion criteria, a total of 2,095 patients undergoing cardiac surgery were included in this study (Figure 1). POD occurred in 415 patients (19.8%) during hospitalization. Baseline characteristics are presented in Table 1. Compared to patients without POD, those who developed POD were significantly older and had a higher proportion of females. While several variables showed statistically significant differences between groups (*p* < 0.01), some differences were minimal from a clinical perspective. For instance, the differences in pH (7.40 vs. 7.39), body temperature (36.38°C vs. 36.61°C), and respiratory rate (16.54 vs. 17.85 breaths/min) all remained within normal physiological ranges, suggesting limited clinical significance. Laboratory tests revealed that POD patients had significantly higher levels of WBC, RDW, AST, LDH, TBIL, Scr, GLU, and BUN. Moreover, NLR, MLR, and PLR were all significantly elevated in the POD group. Regarding comorbidities, POD patients showed significantly higher rates of stroke, congestive heart failure, respiratory failure, and atrial fibrillation, but lower rates of hypertension, coronary artery disease, and dyslipidemia. Regarding surgical types, patients with POD had lower rates of coronary artery bypass grafting (42.17% vs. 59.70%, *p* < 0.001), but higher rates of valve surgeries and thoracic aorta replacement (all *p* < 0.01). No significant differences were observed in other clinical characteristics between the two groups (all *p* > 0.01).

**Table 1:** Baseline characteristics of patients.

**POD**	**No**	**Yes**	***P-*value**
*N, n*	1,680	415	
Age (year), mean ± SD	66.73 ± 11.54	68.48 ± 13.12	0.007
Sex, *n* (%)			< 0.001
Male	1,221 (72.68%)	260 (62.65%)	
Female	459 (27.32%)	155 (37.35%)	
Race, *n* (%)			0.600
White	1,204 (71.67%)	287 (69.16%)	
Asian/black/Hispanic	168 (10.00%)	45 (10.84%)	
other	308 (18.33%)	83 (20.00%)	
BMI (kg/m^2^), mean ± SD	29.14 ± 5.54	28.80 ± 5.66	0.262
SBP (mmHg), mean ± SD	113.74 ± 17.96	113.26 ± 19.09	0.632
DBP (mmHg), mean ± SD	59.40 ± 11.84	59.03 ± 13.22	0.577
Heart rate (beats/min), mean ± SD	80.49 ± 12.68	85.34 ± 16.36	< 0.001
Respiratory rate (breaths/min), mean ± SD	16.54 ± 4.54	17.85 ± 5.59	< 0.001
Body Temperature (°C), mean ± SD	36.38 ± 0.71	36.61 ± 0.78	< 0.001
pH, mean ± SD	7.40 ± 0.05	7.39 ± 0.07	< 0.001
RBC (× 10^12^/L), mean ± SD	3.59 ± 0.79	3.55 ± 0.79	0.337
HB (g/L), mean ± SD	10.76 ± 2.32	10.64 ± 2.35	0.325
RDW (%), mean ± SD	13.90 ± 1.67	14.71 ± 1.68	< 0.001
Hematocrit (g/L), mean ± SD	32.61 ± 6.74	32.13 ± 6.94	0.195
WBC (× 10^9^/L), mean ± SD	10.56 ± 5.54	11.44 ± 5.27	0.004
PLT (× 10^9^/L), mean ± SD	177.92 ± 73.56	186.04 ± 92.49	0.345
ALT (U/L), mean ± SD	36.62 ± 119.29	59.59 ± 175.37	0.111
AST (U/L), mean ± SD	55.72 ± 326.21	98.63 ± 330.96	< 0.001
LDH (U/L), mean ± SD	281.51 ± 338.31	396.43 ± 683.02	< 0.001
ALP (U/L), mean ± SD	78.14 ± 41.37	85.89 ± 74.35	0.225
TBIL (U/L), mean ± SD	0.68 ± 0.77	1.04 ± 1.49	< 0.001
Albumin (g/L), mean ± SD	3.68 ± 0.44	3.43 ± 0.60	< 0.001
Scr (umol/L), mean ± SD	1.19 ± 1.08	1.51 ± 1.54	< 0.001
GLU (mg/dL), mean ± SD	7.46 ± 1.28	7.79 ± 1.69	< 0.001
BUN (mg/dL), mean ± SD	21.44 ± 13.26	27.99 ± 19.62	< 0.001
PLR, mean ± SD	142.64 ± 124.15	169.05 ± 152.25	< 0.001
NLR, mean ± SD	5.78 ± 4.36	7.83 ± 6.15	< 0.001
MLR, mean ± SD	0.32 ± 0.31	0.41 ± 0.38	< 0.001
Hypertension, *n* (%)	903 (53.75%)	194 (46.75%)	0.011
Diabetes, *n* (%)	635 (37.80%)	155 (37.35%)	0.866
Stroke, *n* (%)	196 (11.67%)	82 (19.76%)	< 0.001
Congestive heart failure, *n* (%)	511 (30.42%)	205 (49.40%)	< 0.001
Coronary artery disease, *n* (%)	1,298 (77.26%)	288 (69.40%)	< 0.001
Respiratory failure, *n* (%)	205 (12.20%)	171 (41.20%)	< 0.001
Dyslipidemia, *n* (%)	1,220 (72.62%)	263 (63.37%)	< 0.001
Atrial fibrillation, *n* (%)	840 (50.00%)	301 (72.53%)	< 0.001
**Surgical types**, ***n*** **(%)**			
Coronary artery bypass grafting	1,003 (59.70%)	175 (42.17%)	< 0.001
Mitral valve repair	29 (1.73%)	14 (3.37%)	0.034
Aortic valve replacement	469 (27.92%)	142 (34.22%)	0.011
Mitral valve replacement	85 (5.06%)	37 (8.92%)	0.003
Thoracic aorta replacement	83 (4.94%)	41 (9.88%)	< 0.001
Others	11 (0.66%)	6 (1.45%)	0.108

BMI, Body Mass Index; RBC, Red Blood Cell; HB, Hemoglobin; RDW, Red Cell Distribution Width; WBC, White Blood Cell; PLT, Platelet; ALT, Alanine Aminotransferase; AST, Aspartate Aminotransferase; LDH, Lactate Dehydrogenase; ALP, Alkaline Phosphatase; TBIL, Total Bilirubin; Scr, Serum Creatinine; GLU, Glucose; BUN, Blood Urea Nitrogen.

### 3.2 Association between NLR, MLR, PLR, and POD risk

Logistic analysis models revealed the associations of NLR, MLR, PLR with POD (Table 2). In the unadjusted Model 1, a significant positive association was observed between NLR and POD (Q4 vs. Q1: OR (95% CI): 2.78 (2.02, 3.83), *p* < 0.001, *P* for trend < 0.001). In Model 2, after adjusting for potential confounders, NLR still showed a positive correlation with POD (Q4 vs. Q1: OR (95% CI): 2.98 (2.16, 4.12), *p* < 0.001, *P* for trend < 0.001). In the fully adjusted Model 3, NLR remained independently related to the increased risk of POD (Q4 vs. Q1: OR (95% CI): 2.16 (1.53, 3.05), *p* < 0.001, *P* for trend < 0.001). When NLR was considered as a continuous variable, we observed that for each unit increase in NLR, the risk of POD increased approximately 8% in Model 1 and Model 2 (*p* < 0.001), and 6% in Model 3 (*p* < 0.001).

**Table 2:** Associations of NLR, MLR, and PLR with POD in logistic analysis model.

**Variables**	**Model 1**		**Model 2**		**Model 3**	
	**OR 95%CI**	***P*** **-value**	**OR 95%CI**	***P*** **-value**	**OR 95%CI**	***P*** **-value**
**NLR**						
Continuous	1.08 (1.06, 1.10)	< 0.001	1.08 (1.06, 1.11)	< 0.001	1.05 (1.03, 1.08)	< 0.001
Q1	Ref	–	Ref	–	Ref	–
Q2	1.56 (1.11, 2.19)	0.012	1.63 (1.16, 2.30)	0.005	1.40 (0.98, 2.02)	0.066
Q3	1.70 (1.22, 2.38)	0.002	1.77 (1.26, 2.49)	0.001	1.51 (1.06, 2.16)	0.024
Q4	2.78 (2.02, 3.83)	< 0.001	2.98 (2.16, 4.12)	< 0.001	1.99 (1.41, 2.84)	< 0.001
*P* for trend	1.08 (1.06, 1.10)	< 0.001	1.08 (1.06, 1.11)	< 0.001	1.24 (1.11, 1.38)	< 0.001
**MLR**						
Continuous	1.97 (1.48, 2.64)	< 0.001	2.14 (1.60, 2.87)	< 0.001	1.39 (1.01, 1.92)	0.043
Q1	Ref	–	Ref	–	Ref	–
Q2	0.91 (0.66, 1.26)	0.566	0.96 (0.69, 1.32)	0.785	0.84 (0.59, 1.18)	0.310
Q3	0.92 (0.67, 1.28)	0.633	0.97 (0.70, 1.35)	0.877	0.82 (0.58, 1.15)	0.244
Q4	1.84 (1.37, 2.47)	< 0.001	2.00 (1.49, 2.70)	< 0.001	1.39 (1.01, 1.92)	0.045
*P* for trend	1.97 (1.48, 2.64)	< 0.001	2.14 (1.60, 2.88)	< 0.001	1.12 (1.01, 1.24)	0.040
**PLR**						
Continuous	1.00 (1.00, 1.00)	< 0.001	1.00 (1.00, 1.00)	< 0.001	1.00 (1.00, 1.00)	0.044
Q1	Ref	–	Ref	–	Ref	–
Q2	1.42 (1.04, 1.96)	0.030	1.41 (1.02, 1.94)	0.036	1.40 (1.00, 1.98)	0.051
Q3	1.43 (1.04, 1.96)	0.029	1.42 (1.03, 1.96)	0.031	1.34 (0.95, 1.91)	0.099
Q4	1.67 (1.22, 2.28)	0.001	1.66 (1.21, 2.27)	0.002	1.36 (0.96, 1.92)	0.086
*P* for trend	1.00 (1.00, 1.00)	< 0.001	1.00 (1.00, 1.00)	< 0.001	1.09 (0.97, 1.21)	0.139

Model 1: Unadjusted.

Model 2: Adjusted for age, sex, and BMI.

Model 3: Adjusted for variables in Model 1 plus HB, pH, Scr, AST, LDH, TBIL, albumin, BUN, hypertension, diabetes, congestive heart failure, coronary artery disease, atrial fibrillation, respiratory failure, types of cardiac surgery.

POD, Postoperative Delirium; MLR, Monocyte-to-Lymphocyte Ratio; NLR; Neutrophil-to-Lymphocyte Ratio; PLR, Platelet-to-Lymphocyte Ratio.

For MLR, the unadjusted Model 1 showed a significant positive association with POD (Q4 vs. Q1: OR (95% CI): 1.84 (1.37, 2.47), *p* < 0.001, *P* for trend < 0.001). This association persisted in Model 2 (Q4 vs. Q1: OR (95% CI): 2.00 (1.49, 2.70), *p* < 0.001, *P* for trend < 0.001) and in the fully adjusted Model 3 (Q4 vs. Q1: OR (95% CI): 1.45 (1.06, 2.00), *p* = 0.022, *P* for trend = 0.020). When analyzed as a continuous variable, each unit increase in MLR was associated with a 97%, 114%, and 51% increase in POD risk in Models 1, 2, and 3, respectively (all *p* < 0.05).

Regarding PLR, the unadjusted Model 1 revealed a significant positive association with POD (Q4 vs. Q1: OR (95% CI): 1.67 (1.22, 2.28), *p* = 0.001, *P* for trend < 0.001). This association remained significant in Model 2 (Q4 vs. Q1: OR (95% CI): 1.66 (1.21, 2.27), *p* = 0.002, *P* for trend < 0.001). However, in the fully adjusted Model 3, the association was attenuated and no longer statistically significant (Q4 vs. Q1: OR (95% CI): 1.34 (0.95, 1.90), *p* = 0.093, *P* for trend = 0.166). When analyzed as a continuous variable, each unit increase in PLR was associated with a slight increase in POD risk in all three models (all *p* < 0.05).

**Figure 2:**
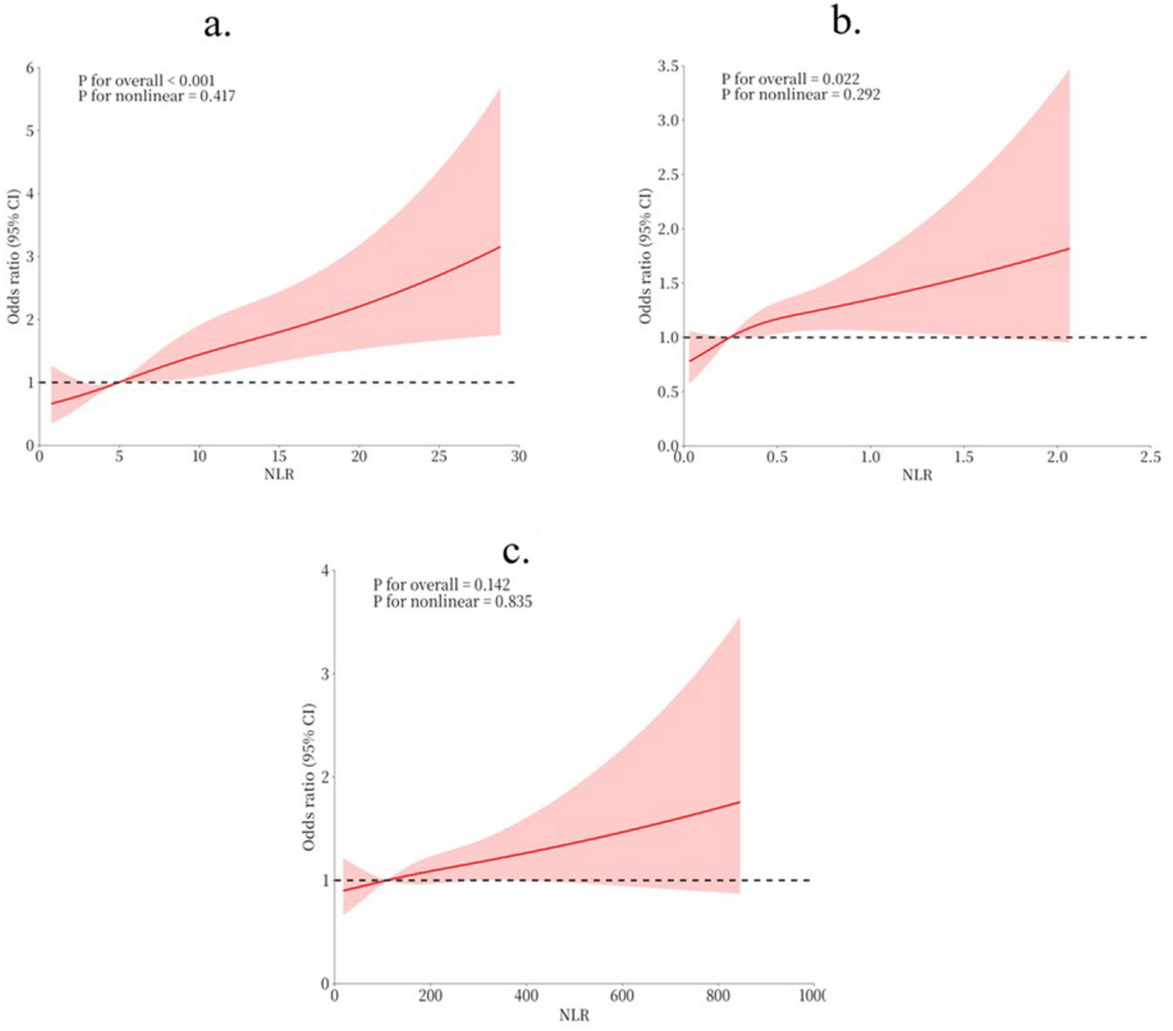
RCS for the relationship between indicators and POD risk. **(a)** RCS for NLR and POD risk; **(b)** RCS for MLR and POD risk; **(c)** RCS for PLR and POD risk.

RCS analysis was conducted to explore the relationships between NLR, MLR, PLR and the risk of POD (Figure 2). The results revealed statistically significant associations between all three markers and POD (all *P* for overall < 0.001). The non-linear components of these relationships did not reach statistical significance (all *P* for non-linear >0.005), suggesting predominantly linear or near-linear associations. The RCS curves demonstrated that as NLR, MLR, and PLR increased, the risk of POD showed a consistent upward trend.

To address the potential impact of severe infections on our findings, we conducted subgroup analyses stratified by WBC count (cutoff value: 10 × 10^9^/L). As shown in Supplementary Table 2, the POD incidence was higher in patients with WBC >10 × 10^9^/L compared to those with WBC ≤ 10 × 10^9^/L (22.61% vs. 17.51%, *p* = 0.004). The associations between inflammatory markers and POD remained significant in both subgroups, particularly for NLR (WBC ≤ 10 × 10^9^/L: OR 1.07, 95% CI 1.03–1.12, *p* < 0.001; WBC >10 × 10^9^/L: OR 1.04, 95% CI 1.01–1.07, *p* = 0.004). Importantly, no significant interactions were observed between WBC count and any of the inflammatory markers (all *p* for interaction >0.05), suggesting our findings were robust regardless of potential underlying infections.

### 3.3 Incremental prognostic value of NLR, MLR, and PLR in POD risk stratification

**Figure 3:**
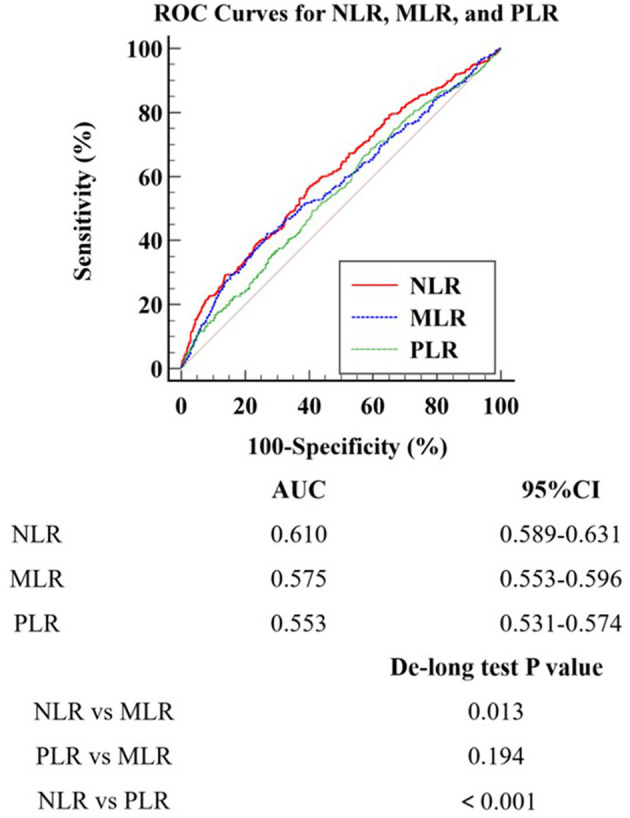
ROC Curves for NLR, MLR, and PLR. *ROC* Receiver Operating Characteristic; *MLR* Monocyte-to-Lymphocyte Ratio; *NLR* Neutrophil-to-Lymphocyte Ratio; *PLR* Platelet-to-Lymphocyte Ratio.

ROC curve analysis was performed to evaluate the predictive performance of NLR, MLR, and PLR for POD (Figure 3). The AUC values for NLR, MLR, and PLR were 0.610, 0.575, and 0.553, respectively. The optimal cut-off values were 5.413 for NLR, 0.357 for MLR, and 91.851 for PLR. De-Long test was conducted to compare the AUC values among these three inflammatory markers. The results revealed that NLR exhibited the strongest predictive ability for POD in patients undergoing cardiac surgery, demonstrating significantly higher predictive value compared to both MLR (*p* < 0.05) and PLR (*p* < 0.05). However, no significant difference was observed in the predictive performance between MLR and PLR (*p* > 0.05).

## 4 Discussion

This study investigated the relationship between inflammatory markers (NLR, MLR, and PLR) and the occurrence of POD in patients undergoing cardiac surgery. Our findings demonstrated that all three indicators were independent risk factors for POD following cardiac surgery, with NLR exhibiting the strongest predictive capacity. NLR showed significantly superior predictive value compared to MLR and PLR. These results provided new tools for assessing the risk of POD after cardiac surgery, with NLR potentially emerging as a simple, cost-effective, and efficient predictive indicator. Given the significant impact of POD on patient outcomes following cardiac surgery, the application of these inflammatory markers may improve the quality of postoperative care for cardiac surgery patients and potentially reduce POD-associated complications and healthcare costs.

The susceptibility of cardiac surgery patients to POD is closely related to their unique pathophysiological characteristics. Firstly, microemboli generated during cardiopulmonary bypass may obstruct cerebral vessels, inducing local inflammation and cerebral edema (19). These microemboli, originating from surgical manipulation or extracorporeal circulation equipment, can lead to cerebral microcirculatory disturbances and neuronal injury (20). Secondly, blood pressure fluctuations and hemodynamic changes during surgery may affect cerebral perfusion, increasing the risk of neurological complications (21). Specifically, episodes of hypotension can cause cerebral tissue hypoxia, while hypertension may induce cerebral edema (22, 23). Furthermore, the systemic inflammatory response following cardiac surgery may promote POD through blood-brain barrier disruption and neuroinflammation (24). Lastly, factors such as postoperative pain and sleep deprivation may also increase the risk of POD (25, 26).

Given the high incidence of POD following cardiac surgery and its complex pathophysiological mechanisms, researchers have increasingly focused on the potential value of inflammatory markers in predicting this complication in recent years. A previous study, also utilizing the MIMIC database, examined the relationship between the MLR and POD, confirming that elevated MLR is a risk factor for POD occurrence, which was consistent with the findings of the present study (15). Building on this foundation, the present study innovatively explored two additional inflammatory indicators: NLR and PLR. The results demonstrated that elevations in both NLR and PLR are also risk factors for POD. More importantly, through comparative analysis, this study found that NLR exhibited superior performance in predicting POD compared to MLR, while PLR's predictive performance was comparable to that of MLR. These findings provide important reference points for selecting the most appropriate predictive indicators for POD in clinical practice following cardiac surgery.

In recent years, an increasing number of studies have revealed a close relationship between inflammatory responses and POD following cardiac surgery. NLR, MLR, and PLR, as inflammatory markers, may participate in the occurrence of POD through multiple mechanisms. Firstly, the unique nature of cardiac surgery, such as the use of cardiopulmonary bypass, can lead to systemic inflammatory response syndrome (SIRS), which is more likely to cause severe inflammatory reactions compared to other types of surgery (27). This widespread inflammatory response can increase blood-brain barrier permeability, allowing more inflammatory factors to enter the central nervous system (28). Neutrophils play a crucial role in this process, not only releasing pro-inflammatory factors but also potentially exacerbating local inflammatory responses and tissue damage through the formation of neutrophil extracellular traps (NETs) (29). Monocytes and macrophages primarily activate microglia by secreting cytokines such as IL-6 and TNF-α, thereby affecting neurotransmitter balance (30). Platelet activation may not only lead to microthrombus formation, but the inflammatory mediators they release can also aggravate endothelial dysfunction (31). This is particularly important in cardiac surgery patients, as they often have underlying cardiovascular diseases and are more susceptible to these effects. In contrast, lymphocytes, especially regulatory T cells, may play a protective role in suppressing excessive inflammatory responses (32). Notably, the trauma of cardiac surgery and the use of anesthetic drugs may further exacerbate these inflammatory responses by affecting immune system function (33). Additionally, the low cardiac output state following cardiac surgery may lead to inadequate cerebral perfusion, further exacerbating neuroinflammation and the risk of POD (34). These mechanisms interact in complex ways, forming a cascade network of inflammatory responses that ultimately leads to neurological dysfunction and the occurrence of POD (35). In cardiac surgery patients, this inflammatory response may be more pronounced because both the surgery itself and cardiopulmonary bypass can trigger strong immune responses (36). These findings provide a theoretical basis for our research results, explaining why NLR, MLR, and PLR can effectively predict the occurrence of POD after cardiac surgery. In particular, we observed that NLR has the strongest predictive ability, which may reflect the central role of neutrophils in postoperative inflammatory responses and the occurrence of POD.

This study also yielded several additional findings. In the multivariate analysis, NLR, MLR, and PLR were all confirmed as independent risk factors, suggesting that they may reflect different aspects of the inflammatory process. Specifically, NLR may more closely reflect the intensity of acute inflammatory responses, MLR may be associated with chronic inflammation and immune regulation, while PLR may indicate platelet activation and involvement of the coagulation system. We observed a potentially linear or near-linear relationship between these three indicators and POD risk, implying that as the values of these indicators increase, the risk of POD correspondingly rises without a clear threshold effect. Based on these findings, we can draw several conclusions. Firstly, NLR was identified as the strongest predictor, indicating that it should be prioritized in preoperative POD risk assessments. Secondly, while we have identified the optimal cut-off values for individual indicators (NLR: 5.413, MLR: 0.357, PLR: 91.851), the development of a comprehensive risk assessment model combining these indicators would require further investigation. This represents an important direction for future research. Lastly, the association between these inflammatory markers and POD risk provides new directions for in-depth investigation of the role of inflammatory responses in POD occurrence, potentially contributing to the development of more targeted prevention and treatment strategies.

Several limitations of this study should be acknowledged. As a retrospective study based on the MIMIC database, we were unable to exclude certain conditions that might affect peripheral blood components, such as perioperative allergic events, hematological disorders, and allogeneic blood transfusions. These factors could potentially influence the inflammatory markers we studied. Furthermore, due to the inherent limitations of retrospective studies, we could not definitively establish whether all delirium cases were directly caused by cardiac surgery, as some cases might be related to other factors such as metabolic disorders, medications, or underlying diseases. Additionally, we were unable to include certain important perioperative parameters such as postoperative pain levels and dynamic changes in hemoglobin and hematocrit during the perioperative period, which might influence the development of POD. This limitation underscores the need for prospective studies with rigorous diagnostic criteria and follow-up assessments to better elucidate the causal relationship between cardiac surgery and postoperative delirium. Additionally, the single-center nature of this study may limit its generalizability. Future prospective, multi-center studies with more rigorous exclusion criteria are needed to validate our findings.

## 5 Conclusion

This study demonstrated that NLR, MLR, and PLR were independent predictors of POD in cardiac surgery patients. Among these inflammatory markers, NLR exhibited the strongest predictive ability for POD. The observed linear relationships between these markers and POD risk further supported their potential as predictive tools. These findings contributed to our understanding of the association between inflammatory markers and POD in the context of cardiac surgery, with NLR emerging as the most promising indicator for risk assessment.

## Data Availability

Publicly available datasets were analyzed in this study. This data can be found here: https://mimic.mit.edu/&lt.
