# Supplementary material for: Comparative analysis of inflammatory markers as predictive markers for postoperative delirium in cardiac surgery patients: an observational study
Source: Front Med (Lausanne). 2025 Apr 1;12:1515940. doi: 10.3389/fmed.2025.1515940 (PMC11996787; doi:10.3389/fmed.2025.1515940)
Supplement: Supplementary file 2 [file Table_2.DOCX]

**Supplementary Table 2.** Subgroup Analysis of the Association Between Inflammatory Markers and POD Stratified by WBC Count

Part A: Baseline Characteristics

| Variables | WBC ≤10×10^9^/L | WBC >10×10^9^/L | P value |
| --- | --- | --- | --- |
| N | 1148 | 947 |  |
| POD, n(%) | 201 (17.51%) | 214 (22.61%) | 0.004 |
| NLR | 5.44 ± 3.68 | 7.08 ± 5.82 | <0.001 |
| MLR | 0.30 ± 0.28 | 0.38 ± 0.38 | <0.001 |
| PLR | 177.90 ± 142.13 | 111.47 ± 104.08 | <0.001 |

Part B: Multivariable Logistic Regression Analysis

| Markers | WBC ≤10×10^9^/L | | WBC >10×10^9^/L | | P for interaction |
| --- | --- | --- | --- | --- | --- |
|  | OR 95%CI | P value | OR 95%CI | P value |  |
| NLR | 1.07 (1.03, 1.12) | <0.001 | 1.04 (1.01, 1.07) | 0.004 | 0.200 |
| MLR | 1.90 (1.14, 3.17) | 0.015 | 1.08 (0.72, 1.62) | 0.702 | 0.090 |
| PLR | 1.00 (1.00, 1.00) | 0.174 | 1.00 (1.00, 1.00) | 0.009 | 0.185 |

Adjusted for variables in Model 1 plus HB, pH, Scr, AST, LDH, TBIL, albumin, BUN, hypertension, diabetes, congestive heart failure, coronary artery disease, atrial fibrillation, respiratory failure, types of cardiac surgery.
